# Diabetes is an independent predictor of survival 17 years after myocardial infarction: follow-up of the TRACE registry

**DOI:** 10.1186/1475-2840-9-22

**Published:** 2010-06-02

**Authors:** Thomas Kümler, Gunnar H Gislason, Lars Køber, Christian Torp-Pedersen

**Affiliations:** 1 Department of Cardiology, Rigshopitalet University Hospital, Blegdamsvej, Copenhagen, Denmark; 2 Department of Cardiology, Gentofte University Hospital, Niels Andersens Vej 65, Copenhagen, Denmark

## Abstract

**Background:**

In patients hospitalized for myocardial infarction, there are limited data examining the long-term prognostic effect of diabetes.

The aim of this study was to systematically evaluate the development of diabetes as an independent long-term prognostic factor after myocardial infarction.

**Methods:**

Prospective follow-up of 6676 consecutive MI patients screened for entry in the Trandolapril Cardiac Evaluation (TRACE) study. The patients were analysed by Kaplan-Meier survival analysis, landmark analysis and Cox proportional hazard models and outcome measure was all-cause mortality.

**Results:**

The mortality in patients with diabetes was 82,7% at 10 years of follow-up and 91,1% at 15 years of follow-up, while patients without diabetes had a mortality of 60,2% at 10 years of follow-up and 72,9% at 15 years of follow-up (p < 0.0001). Landmark analysis continued to show prognostic significance of diabetes throughout the duration of follow-up. Multivariable Cox proportional-hazards model showed that the hazard ratio for death in patients with diabetes overall was 1.47 (95% confidence intervals (CI) 1.35-1.61) and varied between 1.19 (CI 1.04-1.37) and 2.13 (CI 1.33-3.42) in the 2-year periods of follow-up.

**Conclusions:**

Diabetes is an important independent long-term prognostic factor after MI and continues to predict mortality even 17 years after index MI.

This underscores the importance of aggressive diagnostic and therapeutic approach in diabetes patients with MI.

## Background

Disturbances in glucose metabolism are frequent in patients with ischemic heart disease, and abnormal glucose tolerance in MI patients is almost twice as common as hypertension and dyslipidemia [1]. The Glucose Metabolism in Patients with Acute Myocardial Infarction (GAMI) study documented a high prevalence of diabetes and abnormal glucose tolerance in patients MI and no known diabetes [2]. Diabetes increases long-term mortality following MI [3,4], and patients requiring glucose-lowering therapy exhibit a cardiovascular risk of the same magnitude as patients without diabetes with a prior myocardial infarction, regardless of gender and diabetes type [5]. As a result, patients with diabetes should receive prophylactic treatment for cardiovascular disease, but many remain undiagnosed thus missing this treatment effect [6]. Moreover, the most recent guidelines recommend that patients with myocardial infarction are screened for diabetes, including an oral glucose tolerance test when necessary, but this recommendation is new and not widely followed [7]. As a result, it is important to know whether diabetes as an important risk factor does not deteriorate over time even when cardiovascular disease is established, underlining the importance of an aggressive approach towards diagnosing diabetes in MI patients. However, data examining the long-term prognostic effect of diabetes after MI are scarce, and there is lack of information in the variation of the prognostic effect of diabetes over time in MI patients.

We undertook a retrospective study of 6676 consecutive patients admitted with myocardial infarction and screened for entry in the TRACE study. The purpose was to systematically evaluate the long-term development of the prognostic importance of diabetes as a risk factor evaluated at the time of the index infarction.

## Methods

### Subject and study design

The Trandolapril Cardiac Evaluation (TRACE) registry has been described in detail previously [8,9]. In brief, the TRACE registry consist of the 6676 MI patients screened for entry in the TRACE study, which was a double-blind, randomized, parallel group, placebo-controlled study of trandolapril versus placebo in patients with left ventricular dysfunction after MI. The study was conducted in 27 centres in Denmark, and participating centres were required to screen consecutive patients admitted with MI 2-6 days after the infarction and provide data on each patient for the registry. The diagnostic criteria of myocardial infarction were chest pain and/or electrocardiographic changes suggestive of ischemia or infarction, accompanied with elevated cardiac enzymes. Left ventricular systolic function was evaluated in a core lab as wall motion index using a 9-segment model and a reverse scoring system. Wall motion index multiplied by 30 approximates left ventricular ejection fraction. The technique has previously been described in detail and validated [10]. Of the screened patients, 1749 (26.2%) were randomised to trandolapril or placebo in the TRACE study.

The TRACE study was approved by all regional ethical committees in Denmark and complies with the 1975 Declaration of Helsinki. Informed consent was obtained from each patient. The study was registered with the National Board of Health and the Danish Data Protection Agency. All participating patients provided informed consent.

### Follow-up data

All Danish citizens are given a unique and permanent person registry number. All deaths in Denmark are registered in the central person registry within 2 weeks and all deaths are confirmed by a death certificate. Follow-up mortality data were provided by a computerized analysis from the Danish Central Personal Registry by 16.06.2008.

### Statistical analysis

The base-line characteristics of the study population were compared with a t-test for continuous variables and a chi-square test for discrete variables. Mortality was analyzed with Kaplan-Meier curves. We used Landmark analyses to illustrate the prognostic significance of diabetes in 2-year intervals. Relative risk for death and the associated 95 percent confidence intervals were calculated as hazard ratios derived from a Cox proportional-hazards regression model. We used stepwise models including increasing number of variables. The models fulfilled the Cox regression model assumptions (linearity of continuous variables, proportional hazards assumption and lack of interaction) unless otherwise specified. Statistical analyses were performed with the Statistical Analysis System, ver. 9.1 (SAS Institute, Cary, NC, USA).

## Results

### Baseline characteristics

A total of 7001 MI's in 6676 patients were reported from May 1990 to August 1992. By the end of follow-up, the mortality was 5231 patients (78.4%). 38 patients (0.56%) were lost to follow-up or emigrated and were censured on the last day they were known to be alive.

Baseline characteristics of the 6668 patients included in our study are listed in table 1. There were missing data on diabetes status on 8 patients. Patients with diabetes were older, had more co-morbidity and risk factors, received more often diuretics but less often thrombolytic therapy, had poorer left ventricular function and were in higher New York Heart Association (NYHA) and Killip class. All the mentioned differences were statistically significant.

**Table 1:** Patient characteristics according to diabetes classification.

	Diabetes (n = 719)	No diabetes (n = 5949)	P Value
Age (SD)	69.5	67.1	<0.0001
Gender. %	41.3	31.5	<0.0001
Women	58.7	68.5	
Men			
Body mass index (SD)	26.7	25.6	<0.0001
Creatinine (mean). μmol/l	117	107	<0.0001
Hypertension. %	36.3	21.0	<0.0001
Angina pectoris %	48.1	35.5	<0.0001
Previous MI. %	32.3	22.3	<0.0001
Heart failure*. %	69.0	51.8	<0.0001
Current smoking. %	36.1	53.4	<0.0001
Thrombolysis. %	27.0	42.5	<0.0001
Previous stroke. %	13.2	7.6	<0.0001
Diuretic treatment. %	64.2	42.3	<0.0001
NYHA. %			
Class I	47.0	58.4	<0.0001
Class II	34.8	28.3	
Class III	6.9	3.9	
Class IV	9.0	6.3	
Killip. %			
Class I	69.5	79.9	<0.0001
Class II	22.0	14.2	
Class III	2.2	2.11	
Class IV	6.3	3.8	
Wall motion index. %			
>1.6	21.1	34.1	<0.0001
1.3-1.6	20.9	23.9	
0.8-1.2	40.1	31.1	
<0.8	8.2	4.7	

SD, standard deviation; NYHA, New York Heart Association;

P-values calculated with the use of chi2-test for discrete variables and t-test for continuous variables.

*History of heart failure and in-hospital heart failure

At the start of the study, there were 156 (2.34%) patients with diabetes treated with diet, 372 (5.58%) with diabetes treated with oral medication, and 140 (2.10%) with patients with diabetes treated with insulin. There were missing data on treatment in 5 patients.

### Analysis of all-cause mortality

**Figure 1:**
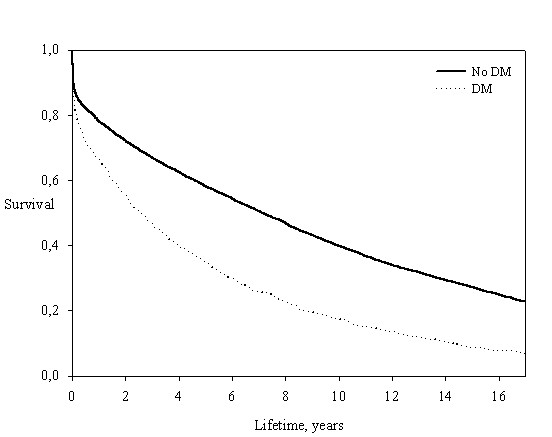
**Unadjusted all-cause mortality stratified by diabetes**.

**Figure 2:**
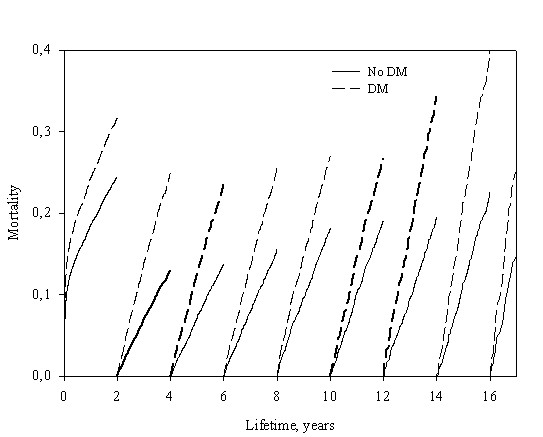
**Landmark analysis of the time dependent prognostic significance of diabetes adjusted for age and gender**.

As expected, patients with diabetes had a significantly higher mortality than patients without diabetes (figure 1, unadjusted analysis). The mortality in patients with diabetes was 82,7% and 91,1% at 10 and 15 years of follow-up while patients without diabetes had a mortality of 60,2% and 72,9% at 10 and 15 years of follow-up (p value < 0.0001 for difference between patients with and without diabetes). To clarify the importance of diabetes as a prognostic factor we performed landmark analysis illustrating survival in diabetics and non diabetics adjusted for age, sex and wall motion index in 2 year intervals after the infarction (Figure 2). The Landmark analysis demonstrated that diabetes continued to have a significant prognostic effect throughout the duration of follow-up.

**Figure 3:**
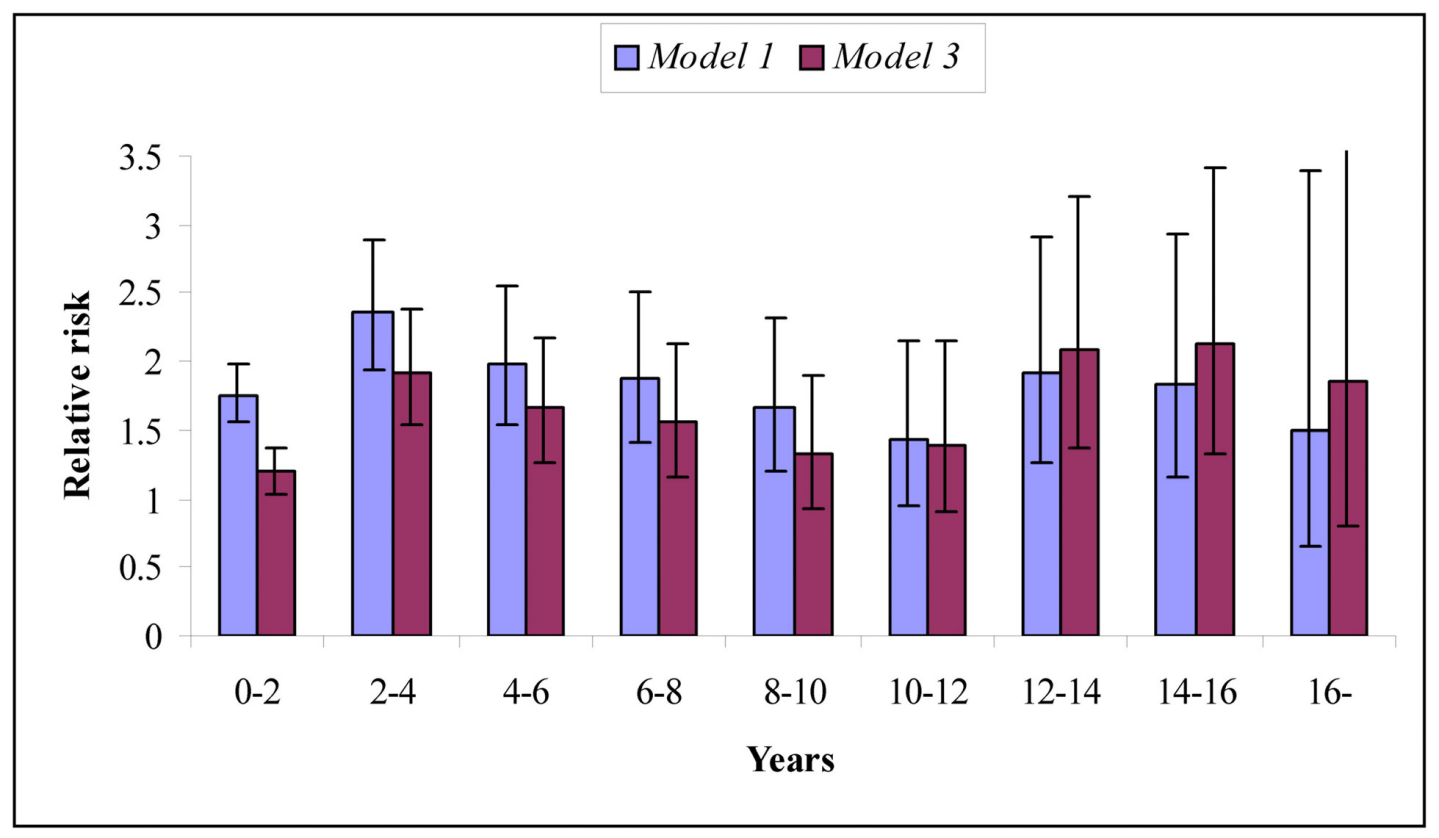
**All cause mortality hazard ratio as a function of follow-up time for Cox proportional hazards model 1 (diabetes) and 3 (all covariates)**.

We constructed Cox proportional-hazards models of total mortality with stepwise addition of covariates (Table 2). Age was a significant prognostic factor throughout the length of follow-up in all models. The hazard ratio (HR) varied between 1.45 (95% Confidence Interval [CI] 1.37-1.54) and 2.14 (CI 1.68-2.71) per 10 years increase in patient age in the model incorporating all covariates. For the entire follow-up period, diabetes was a significant prognostic factor, hazard ratio 1.47 (CI 1.35-1.61) adjusted for all covariates. Greater significance of diabetes reflected in a higher hazard ratio was observed in the end of follow-up, however this did not reach statistical significance in the last period of follow-up, most likely as a result of lack of statistical power since only few patients with diabetes were still alive at this time. Relative risks in the model without covariates (model 1) and with all covariates (model 3) in each of the 2-year periods of follow-up are illustrated in figure 3. HR for diabetes varied between 1.19 (CI 1.04-1.37) and 2.13 (CI 1.33-3.42) in the fully adjusted models and reached significance in most of the 2-year intervals. The hazard ratio associated with male gender was between 0.99 (CI 0.89-1.10) and 1.64 (CI 1.27-2.12) and did not reach statistical significance in most of the 2-year interval. Overall, male gender was associated with a small, but significant increase in risk of death, hazard ratio 1.11 (CI 1.04-1.18).

**Table 2:** Proportional hazards models of mortality as a function of time

	0-2 years		2-4 years		4-6 years		6-8 years		8-10 years		10-12 years		12-14 years		14-16 years		+16 years	
**Variables**	**RR**	CI*	**RR**	CI*	**RR**	CI*	**RR**	CI*	**RR**	CI*	**RR**	CI*	**RR**	CI*	**RR**	CI*	**RR**	CI*

**Model 1**																		
Diabetes	1.76	1.56-1.98	2.36	1.93-2.89	1.98	1.54-2.55	1.88	1.41-2.50	1.66	1.19-2.32	1.43	0.96-2.15	1.92	1.26-2.91	1.84	1.15-2.93	1.50	0.66-3.40
**Model 2**																		
Male gender	1.03	0.94-1.13	1.36	1.15-1.60	1.02	0.85-1.22	1.23	1.01-1.49	1.19	0.97-1.46	1.60	1.26-2.03	1.05	0.82-1.34	1.04	0.80-1.35	1.05	0.71-1.55
Age**	1.86	1.77-1.95	2.02	1.86-2.20	1.90	1.72-2.08	2.08	1.90-2.30	2.08	1.88-2.30	2.12	1.90-2.39	2.14	1.88-2.43	1.99	1.72-2.28	2.12	1.71-2.62
Diabetes	1.57	1.39-1.77	2.21	1.81-2.71	1.87	1.46-2.41	1.86	1.39-2.48	1.67	1.20-2.33	1.49	0.99-2.24	1.97	1.30-3.00	2.00	1.26-3.19	1.69	0.74-3.85
Model 3***																		
Male gender	0.99	0.89-1.10	1.29	1.08-1.54	1.03	0.85-1.24	1.31	1.07-1.61	1.08	0.87-1.34	1.64	1.27-2.12	1.05	0.80-1.37	1.11	0.82-1.49	0.99	0.64-1.53
Age**	1.45	1.37-1.54	1.71	1.55-1.88	1.69	1.52-1.86	1.88	1.69-2.10	1.90	1.69-2.10	1.99	1.74-2.24	1.97	1.71-2.28	1.90	1.64-2.20	2.14	1.68-2.71
Diabetes	1.19	1.04-1.37	1.92	1.54-2.38	1.66	1.27-2.17	1.57	1.16-2.12	1.34	0.94-1.90	1.40	0.91-2.16	2.09	1.37-3.20	2.13	1.33-3.42	1.86	0.80-4.32

*95% confidence interval

**Risk ratio associated with an increase in age of 10 years.

***Other covariates in model 3: Body mass index, previous AMI, angina pectoris, creatinine, congestive heart failure, diabetes mellitus, wall motion index, systemic hypertension, thrombolytic therapy.

## Discussion

This study describes the long-term prognostic significance of diabetes in a large population of consecutive patients with MI. With a follow-up of 17 years, we found that diabetes continued to represent a strong independent prognostic factor for all-cause mortality. This is a plausible result from a biological point of view as diabetes is a progressive and chronic disease.

Previous studies has documented that diabetes is a well described predictor for an adverse outcome following myocardial infarction [11]. Even patients with pre-diabetic conditions have an elevated risk of cardiovascular disease [12], and it seems a progressive threshold between blood glucose and cardiovascular risk exist, also below the diabetic threshold [13]. Our results expand on these findings by documenting continued prognostic importance of diabetes with a follow-up period much longer than any other study of this subject. In addition, our results support a previous analysis of some of the same patients suggesting that the magnitude of the prognostic effect of diabetes may increase with time [4]. These new results combined with the knowledge of many undiagnosed and thus untreated patients with diabetes underscores the necessity of an aggressive approach towards diagnosing diabetes in MI patients.

It has been suggested that the glucometabolic state at the time of admission for MI confers long-term prognostic information in patients with and without diabetes [14], which would mean that elevated blood glucose level at admission with MI is not only a negligible expression of acute physiological stress. This hypothesis is supported by the observation that both in patients with and without diabetes, blood glucose concentration at the time of admission is related to the risk of death in patients admitted with MI [15]. These studies underscore the close relationship between abnormal glucose metabolism and ischemic heart disease which could be explained by more extensive atherosclerosis in diabetic patients, a theory supported by some, but not all studies [16,17]. Another theory hypothesizes that metabolic disturbance could have an adverse effect in the process of myocardial infarction. This was tested in trials where MI patients with diabetes where treated intensively with insulin to better glycemic status. One trial showed an improvement in long-term mortality but no effect on short term mortality [18]. Another trial failed to show any benefit of the intervention, but epidemiological analysis confirmed that glucose level was a strong predictor of long-term mortality, suggesting that glucose control is important [19]. A smaller study showed no difference in mortality between intervention and control group [20]. In conclusion, no definitive effect of intensive treatment has been documented.

Of great interest is whether long-term intensive diabetes treatment after discharge can lower the risk of developing new cardiovascular events. Epidemiological studies and meta-analyses have shown a clear relationship between haemoglobin A1C and the frequency of cardiovascular disease [21,22], and large studies have suggested beneficial effects of intensive glucose regulation on the risk of cardiovascular events, but such an effect has not been clearly defined [23,24]. Recently, 3 large randomized clinical studies failed to document an effect of intensive glucose regulation on cardiovascular events [25-27].

Despite the lack of evidence for the effect of intensive glucose regulation on cardiovascular events, we believe there are several reasons why a more aggressive approach to diagnosing diabetes is warranted. First, earlier diagnoses would have great significance for risk stratification by identifying patients at high risk. Second, initiation of earlier and more widespread medical treatment would be possible. Improved prognosis would result, since the effect of intensive glucose regulation on micro vascular complications are well documented [23,24], as is multiple risk factor intervention [28]. Third, the lack of documented effect of intensive glucose regulation on cardiovascular events in randomized trials could have other explanations. The effect of intensive glucose treatment could be modest in comparison with the well documented effect of treating other risk factors and thus difficult to document unless in longer trials with higher event rate. The randomized trials have compared usual care diabetes regulation with intensive regulation, but it is still possible that cardiovascular events could be prevented when comparing poor regulation with good regulation. The population included in randomized trials had greater presence of atherosclerosis suggested by long diabetes duration, multiple risk factors or known cardiovascular disease. Subset analyses have shown benefit of intensive diabetes regulation in patients with lesser presence of atherosclerosis [29]. Current strategies for regulation of diabetes could have opposing effects on risk of cardiovascular disease.

Our study has some limitations that need to be acknowledged. When patients were screened for entry into the TRACE study, thrombolytic therapy was administered routinely to ST-elevation MI patients. The standard treatment today in Denmark is percutaneous coronary intervention. Moreover, almost all MI patients today receive clopidogrel, statins and beta-blockers. Some receive spironolactone or eplerenone. New pharmacological treatment combinations are today available to patients with diabetes. The most recent guidelines state that patients with diabetes and albuminuria or hypertension should be treated with an ACE inhibitor or an angiotensin receptor blocker, even when the left ventricular systolic function is normal [30]. This was not the case at the start of the TRACE study, probably resulting in an under-treatment of patients with diabetes during the TRACE study period and the start of the follow-up. These changes in the management of patients with MI and diabetes could significantly influence our results. However, a multivariable analysis from 1975 to the end of 2003 reveal only slight improvement in post discharge survival after MI [31], but this is probably explained by a lack of multivariate adjustment for MI complications, medical and interventional treatment, as the survivors had more aggressive treatment and fewer complications. An important point is that we do not have information regarding the medical treatment of patients with and without diabetes during the follow-up period and thus can not analyse potential differences. Since a number of patients without diabetes at the time of the infarction have developed diabetes later, the observed difference between patients with and without diabetes is smaller than the actual difference.

Many data shows that the diagnosis and treatment of diabetes in cardiovascular patients is not adequately done. New guidelines recommend oral glucose tolerance test in patients with myocardial infarction but this is not formally implemented. Our study is important as it underlines the importance of pinpointing the effect of diabetes as a long-term prognostic factor in MI patients. To our knowledge, this is the first study to systematically evaluate the time-dependent development of diabetes as a long-term prognostic factor in consecutive MI patients with a follow-up of up to 17 years. The population described here was recruited from 27 Danish hospitals with regional patient uptake and can be considered to be representative of patients with MI admitted alive in a western industrialized country. By documenting the continued prognostic importance of diabetes as a prognostic factor in patients with ischemic heart disease, our results supports an aggressive approach towards diagnosing diabetes.

## Conclusions

Diabetes is a long-term negative prognostic factor in MI patients that continues to influence prognosis for up to 17 years after MI. This underscores the importance of an aggressive diagnostic approach towards diabetes. The presence of diabetes identifies MI patients at high-risk, whom are candidates for continued aggressive medical therapy.

## Competing interests

The authors declare that they have no competing interests.

## Authors' contributions

TK was responsible for all stages of the study with the exception of data collection and follow-up: Analysis of data, analysis of the literature, and preparation of the manuscript.

GG supervised the entire study, participated in follow-up, analysis and interpretation of data, and revised the manuscript.

LK supervised the entire study, participated in follow-up, analysis and interpretation of data, and revised the manuscript.

CTP conceived the idea for the study, supervised the study, collected data, analyzed data and revised the manuscript.

All authors have seen and approved the manuscript.
